# Environmental Stability of Additively Manufactured Thermoplastic Polyamide Composites

**DOI:** 10.3390/polym15163385

**Published:** 2023-08-12

**Authors:** Michael J. Imburgia, Jessica L. Faust, Johan Ospina Buitrago, Rachael E. Enfield, Joseph D. Roy-Mayhew

**Affiliations:** Markforged, Inc., Waltham, MA 02451, USA

**Keywords:** fused filament fabrication, carbon fiber reinforcement, polyamide, UV stability, environmental conditioning, mechanical properties

## Abstract

As the adoption of additive manufacturing technologies for end-use parts continues to progress, the evaluation of environmental durability is essential for the qualification of manufactured articles in industries such as automotive, aerospace, and electrical. This study explores the effects of UV and water-spray exposure on the mechanical properties of an additively manufactured polyamide 6 blend reinforced with short carbon fiber and continuous carbon fiber. Fused-filament-fabrication-printed test samples were exposed to a Xenon-arc UV source following ASTM G155 Cycle 1 conditions for a duration of 1000 h. Tensile, flexural, and Izod impact tests were performed on exposed and unexposed test samples. While Exposed tensile and flexural samples maintained their strength (84–100% and 88–100%, of Control samples, respectively), Izod impact strength increased (104–201% of Controls). This study also examines the influence of coatings and finds that samples coated with Krylon^®^ Fusion All-In-One^®^ and JetFlex^®^ Polyurethane Primer maintain similar mechanical properties and exhibit a better visual appearance as compared to uncoated, exposed samples.

## 1. Introduction

In recent years, additive manufacturing has begun to shift away from a technology predominantly used for prototyping to one that aims to create functional end-use parts for high-demand applications [1]. A key first step in this shift has been to understand the fundamentals of additive manufacturing processes, such as the physics of bead-to-bead welding in fused filament fabrication (FFF) technologies, in order to optimize manufactured part performance [2,3,4]. As this shift progresses, it is important to measure the key end-use properties such as mechanical strength and dimensional stability at the time of manufacture, but it is equally important to understand the evolution of these properties over the lifetime of the part [5]. Environmental factors such as temperature, moisture, and ultraviolet light (UV) exposure on 3D-printed materials are less understood but vital to predicting long-term part success [6,7,8,9]. Radiation and chemical exposure are known to degrade plastic parts, and sunlight (UV) and rain (water) are no exception. Plastic parts made for exterior applications undergo additional use testing and are often specifically formulated to meet performance requirements in these conditions. For example, evaluating the effects of UV exposure and water spray is useful for the qualification of manufactured articles in the automotive, aerospace, and electrical industries, among others [10,11].

While many approaches to quantifying the resistance of materials to environmental degradation exist, a straightforward method is to leave a part in the anticipated environment and track part performance over the desired lifetime. This is generally not feasible due to engineering design timelines; thus, accelerated aging methods are often used to evaluate outdoor suitability. A common method used in the plastics industry is to expose test coupons to an energy source mimicking the irradiance of the solar spectrum for a set period of time, while incorporating an element of moisture (e.g., a water spray).

UV-focused energy sources used include Carbon-arc, Xenon-arc, and QUV; each have different distributions of wavelength and intensity, also known as spectral power distribution [12]. A Xenon-arc source with a daylight filter was chosen for this study as it best represents the spectral power distribution of solar radiation [12,13]. Moisture sources and conditions used to evaluate outdoor use of plastic parts include water spray and water immersion [12,13,14]. Hygroscopic polymers, such as polyamides, are susceptible to both hydrolytic degradation and property reduction in the presence of moisture; thus, the effect of moisture is an important aspect to evaluate in environmental exposure testing [14,15,16,17,18,19,20]. Methods which specify exposure details for Xenon-arc sources, such as irradiance, wavelength, moisture conditions, and cycle duration, include ASTM G155 and ISO 4982-2 [21,22]. These methods are utilized in conjunction with industry standards to qualify plastics for use in outdoor environments; for example, the UL 746C f1/f2 outdoor suitability of plastics program requires that a tested material retain 70% of the Control tensile or flexural strength, retain 70% of the Control impact strength, and maintain the flame performance of the Control after 1000 h of ASTM G155 Cycle 1 Xenon-arc exposure [11,21]. Although studies evaluating the UV stability of plastics manufactured using conventional methods (e.g., injection molding) are common, very few additive manufacturing studies addressing the UV stability of printed materials exist [6,7,8,9].

Herein, the effects of UV and water-spray exposure on the mechanical properties of additively manufactured test coupons using both micro carbon fiber (“short fiber”)-filled polyamide 6 (PA6) blends and continuous fiber-reinforced PA6 blends are explored in order to aid in the design and function of end-use parts in adverse environments. In addition, the use of protective coatings has been shown to preserve additively manufactured part properties during accelerated aging [23]. Therefore, samples were coated with UV-stabilizing paints such as Krylon^®^ Fusion All-In-One^®^ (Sherwin-Williams, Cleveland, OH, USA) and JetFlex^®^ White Polyurethane Primer (Sherwin-Williams, Cleveland, OH, USA) to reduce the effect of UV degradation and preserve mechanical properties.

## 2. Materials and Methods

### 2.1. Materials and Printers

The 3D printing materials and printers used in this study were provided by Markforged (Waltham, MA, USA). The short fiber (e.g., discontinuous) composite filaments used for sample fabrication were Onyx^®^ and Onyx^®^ FR, both PA6 blend plastics filled with micro carbon fiber (~100 μm length, ~7 μm diameter). The continuous reinforcement fibers used for sample fabrication were Continuous Fiber—Carbon (CCF) and Continuous Fiber—Carbon FR (CCF FR-A). The densities of Onyx, Onyx FR, CCF, and CCF FR-A are 1.18, 1.22, 1.44, and 1.46 g/cm^3^, respectively. The selected coatings used were Krylon Fusion All-In-One (Primer + Paint; for use with Onyx) and JetFlex White Polyurethane Primer (for use with Onyx FR). All parts were printed using Markforged’s Eiger™ software on two Markforged X7 Industrial Printers using slicer version v 3.27.2 LTS.

### 2.2. Print Parameters and Test Coupon Preparation

**Figure 1:**
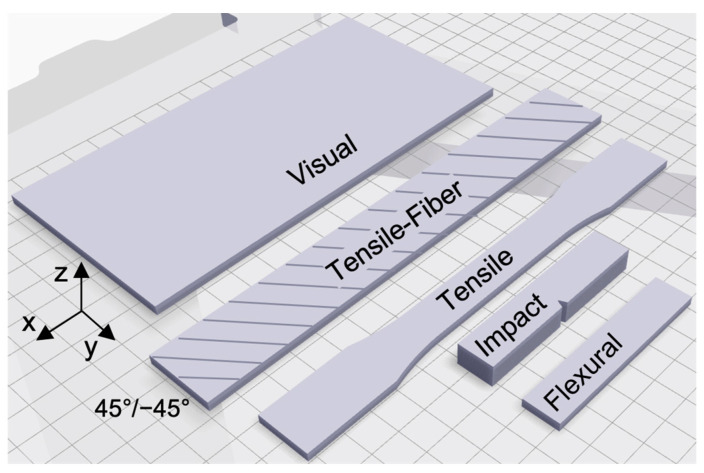
Schematic showing sample types and print orientation with respect to the build plate coordinates.

Samples of test coupons were printed with the same plastic material nozzle size (0.4 mm orifice diameter), plastic material nozzle temperature (275 °C), and default plastic settings for solid infill (96% density, 45°/−45° plastic infill orientation, 0.125 mm layer height, 2 walls, and 4 floors). As shown in Figure 1, the print orientation of 45°/−45° is the angle relative to the long axis (x-axis) of the printed test samples.

The types of samples and their dimensions are described in Table 1.

**Figure 2:**
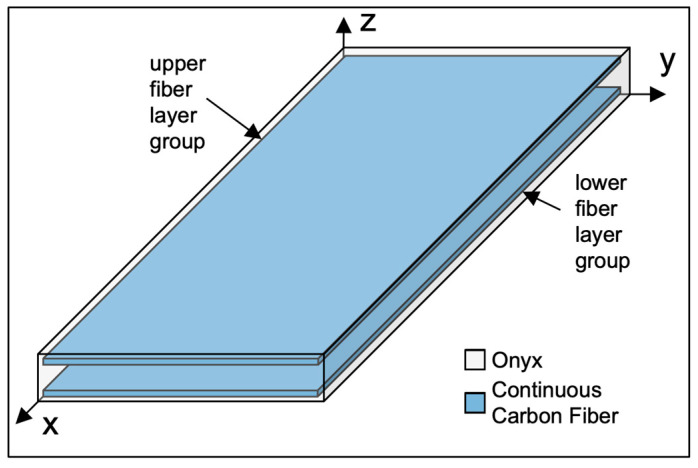
Schematic of continuous fiber composite sample showing an approximate volume fraction of continuous fiber material (Continuous Carbon Fiber, blue) compared to short fiber material (Onyx, gray), the “upper” and “lower” fiber layer groups, and sample orientation.

Short fiber composite test coupons of tensile, Izod impact, flexural, and visual samples were prepared using Onyx, Onyx with a Krylon Fusion All-in-one coating (Onyx-KF), Onyx FR, and Onyx FR with a JetFlex coating (Onyx FR-JF). Continuous fiber composite test coupons of tensile fiber, Izod impact, and flexural were prepared using Onyx with CCF (Onyx-Fiber) and Onyx FR with CCF FR-A (Onyx FR-Fiber). Table 2 outlines the fiber routing details for Onyx-Fiber and Onyx FR-Fiber samples. Figure 2 shows a representation of continuous fiber layering in an example part.

Each fiber sample contains eight print layers of continuous fiber, separated into two groups of four described as the lower and upper layer groups in Table 2 and shown schematically in Figure 2. The Fiber Infill Orientation describes the direction of continuous fiber in each of the fiber layer groups. In the case of Izod impact, each of the four fiber layers within the layer group had a different orientation, thus the four different angles. The Fiber Layer Numbers describes the print layers that contain isotropic continuous fiber infill.

### 2.3. Environmental Exposure Conditions

An Atlas Model Ci4000 Xenon Arc Weather-Ometer^®^ (AMETEK, Inc., Berwyn, PA, USA) was used for UV exposure, performed at a Nationally Recognized Testing Laboratory (NRTL) facility. Exposure conditions following ASTM G155 Cycle 1 were chosen with a daylight filter, irradiance of 0.35 W/(m^2^ × nm) at a wavelength of 340 nm, and an exposure cycle of 102 min light at 63 °C black panel temperature at 50% relative humidity (RH) and 18 min light and water spray (air temperature not controlled). “Exposed” tensile, flexural, and impact strength samples all experienced 1000 h under the aforementioned conditions. Two visual sample plaques for each material were placed in the chamber. One sample of each short fiber composite material was removed from the chamber at 500 h and a second sample of each material was removed at 1000 h.

### 2.4. Sample Drying

Samples were removed from printers within 30 min of print completion and stored in a dry box of 9.5 L volume with 2 desiccant bags (2 Unit Pak, Desiccare, Inc., Las Vegas, NV, USA) to prevent additional moisture uptake. After all samples were printed and stored, they were dried for 48 h at 75 °C under vacuum. Control samples were stored in moisture proof bags without desiccant to preserve the dry state prior to mechanical testing. Exposed samples were stored in moisture proof bags without desiccant until placement into the environmental chamber. After the 1000 h of environmental exposure, the Exposed samples were dried in an oven for 48 h at 75 °C under vacuum to remove residual moisture absorbed from the environmental chamber.

### 2.5. Sample Coating Preparation

Coated samples include Onyx with a Krylon Fusion All-in-one coating (Onyx-KF) and Onyx FR with a JetFlex coating (Onyx FR-JF). Onyx samples were coated with two coats of Krylon Fusion All-in-one Matte Black spray paint only on the anticipated exposure surface of test samples, waiting 30 min between coats. Coats were applied using the as-received spray can from Krylon. Onyx FR samples were coated with two coats of JetFlex White Polyurethane Primer. JetFlex primer was applied using a 3M Accuspray system (3M, St. Paul, MN, USA), a 1.8 mm nozzle, and 20 psi dynamic inlet pressure. The JetFlex primer required dilution with a JetFlex Reducer Solvent Base (approximately 50 wt%) in order to optimize paint spraying finish. The second coat was applied only after the first coat was dry to the touch, approximately 5–10 min after initial application. Coated samples were exposed to ambient conditions within a laboratory fume hood for the 24 h of the required coating curing process. These samples were then dried for an additional 48 h at 75 °C under vacuum.

### 2.6. Moisture Verification

Moisture measurements were collected with an Ametek Brookfield Computrac Vapor Pro XL moisture analyzer (AMETEK, Inc., Berwyn, PA, USA). Moisture levels of 3.6 mm thick Onyx-KF and Onyx FR-JF plaque samples were measured prior to exposure testing. Moisture levels of 8.1 mm thick Onyx Izod impact samples were measured after Izod impact testing.

### 2.7. Mechanical Testing

Tensile, flexural, and impact samples were tested for this study. For tensile, flexural, and Izod impact samples, two sets of six samples each were printed on two Markforged X7 printers (Printers A and B). Three samples from each of Printers A and B were used for Control sample sets, and the remaining samples were used for Exposed sample sets. Five samples were used for each test and the values reported are mean and ±1 standard deviation (SD) from the mean. The aim of using two printers was to reduce the effects of printer-to-printer and material lot variability on the average mechanical properties reported in this study.

#### 2.7.1. Tensile Testing

Tensile testing was performed on short fiber composite samples using the ASTM D638 standard as guidance [24]. All pre- and post-exposure tests were performed on a 3369 Universal Testing Machine frame (Instron, Norwood, MA, USA)) using wedge grips, a crosshead speed of 2 mm/min, and test conditions of 23 °C ± 2 °C/50% ± 10% RH.

Tensile testing was performed on continuous fiber composite samples using the ASTM D3039 standard as guidance [25]. Testing was performed at a NRTL facility using an Instron 5985 frame, a grip pressure of 5.5 MPa, crosshead speed of 2 mm/min, and test conditions of 23 °C ± 2 °C/50% ± 10% RH.

#### 2.7.2. Flexural Testing

Flexural testing was performed using the ASTM D790 standard as guidance [26]. All pre- and post-exposure tests were performed on an Instron 3369 Universal Testing Machine frame. The span during testing was 50.4 mm. Short fiber composite samples were tested at 11.5 mm/min. The maximum flexural stress reported for short fiber composite samples was either a maximum flexural stress if below 5% flexural strain or a flexural stress at 5% flexural strain, following the guidance of ASTM D790. Continuous fiber composite samples were tested at a crosshead rate of 1.15 mm/min. All continuous fiber composite samples broke within 5% flexural strain.

#### 2.7.3. Impact Testing

Impact testing was performed using the ASTM D256 standard for Izod impact as guidance [27]. Izod impact beams were printed with an existing notch geometry (0.4 mm depth). Testing was performed by a NRTL facility on a Tinius Olsen Model 892 Impact Tester (Tinius Olsen TMC, Horsham, PA, USA) using a 2 ft-lb pendulum.

### 2.8. Caprolactam Extraction Testing

Onyx samples used during tensile testing were stored in moisture-proof bags after testing. Upon removal, 0.240 ± 0.002 g samples of each dog bone were cut from the gauge section and weighed. The mass of each sample was recorded with a high-precision laboratory scale (AG204 DeltaRange, Mettler Toledo, Columbus, OH, USA). Samples were then pressed into a thin film using a heated Carver press set at 230 °C and 0.5 MPa of pressure. Samples were pressed into films in order to maximize extraction surface area. After 1 min, samples were removed and placed into 20 mL scintillation vials. Vials were filled with 20 mL of 99% denatured ethanol (Techsol A, PTI Process Chemicals, Ringwood, IL, USA) and placed into a 70 °C oven for 22 h. Upon removal, samples were rinsed with fresh denatured ethanol and placed into empty vials. Samples were dried for 6 h at 110 °C under vacuum. Once allowed to cool to room temperature, samples were weighed and compared to their prepared weight to calculate percent loss of caprolactam.

## 3. Results and Discussion

Mechanical behaviors as a function of environmental exposure are important for end-use part design in a wide variety of end-use applications. Metrics often used for mechanical property retention include tensile strength, flexural strength, and impact strength. For instance, a UL 746C f1/f2 outdoor suitability for rating plastics requires a tested material retain 70% of the Control tensile or flexural strength, retain 70% of the Control impact strength, and maintain the flame performance of the Control after 1000 h of ASTM G155 Cycle 1 Xenon-arc exposure [21]. Inspired by these industrial standards, this study compares the mechanical properties, specifically tensile, flexural, and impact, and visual properties before and after accelerated environmental exposure using ASTM G155 Cycle 1 Xenon-arc exposure. We first explore the *Tensile Properties of Short Fiber Composites* by comparing tensile data from before and after exposure. We then examine the *Tensile Properties of Continuous Fiber Composites*. This section is followed by the *Flexural Properties of Short Fiber and Continuous Fiber Composites* and *Impact Strength of Short Fiber and Continuous Fiber Composites.* We then evaluated the *Visual Differences between Unexposed and Exposed Samples*. The study concludes with the *Role of Residual Caprolactam in Stabilization of Onyx.*

### 3.1. Tensile Properties of Short Fiber Composites

**Figure 3:**
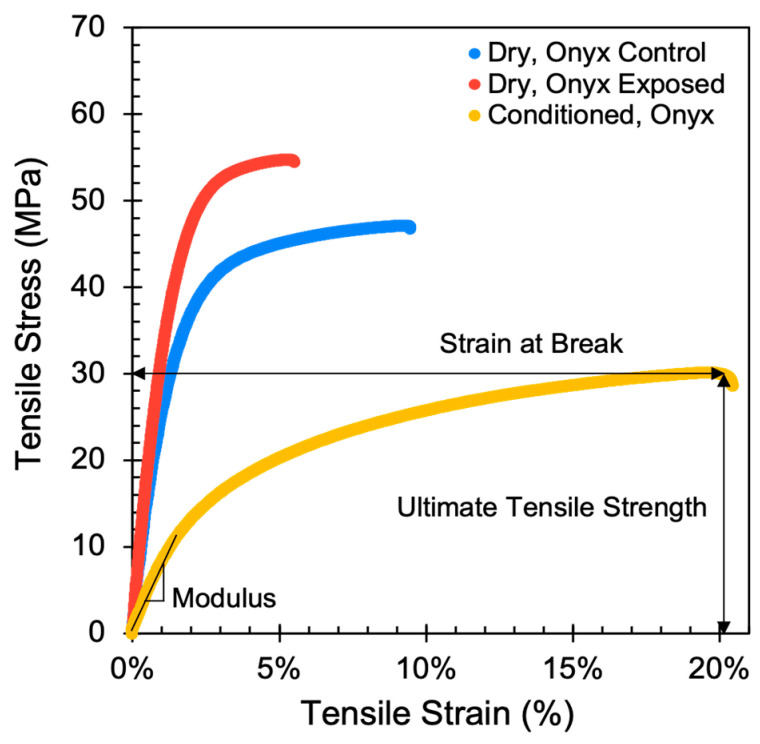
Tensile stress–strain curves of Onyx with 45°/−45° infill at various conditions. Exposed refers to samples tested after 1000 h of Xenon-arc and water-spray exposure. Control refers to unexposed samples. Dry refers to samples dried for 48 h at 75 °C in a vacuum oven after printing. Conditioned refers to samples dried then conditioned at 52 ± 4% RH for 44 ± 2 h. The Modulus, Ultimate Tensile Strength (UTS), and Strain at Break are annotated on the plot.

Tensile properties were measured to understand how various Markforged materials and third-party coatings evolve after environmental exposure. Properties of interest include ultimate tensile strength (UTS), tensile modulus, and strain at break. Figure 3 highlights representative stress–strain curves of Markforged Onyx comparing different sample preparation conditions. The conditions include a moisture-conditioned FFF tensile test coupon, a dry tensile test coupon (the control condition in this study), and an environmentally exposed dry tensile test coupon.

**Figure 4:**
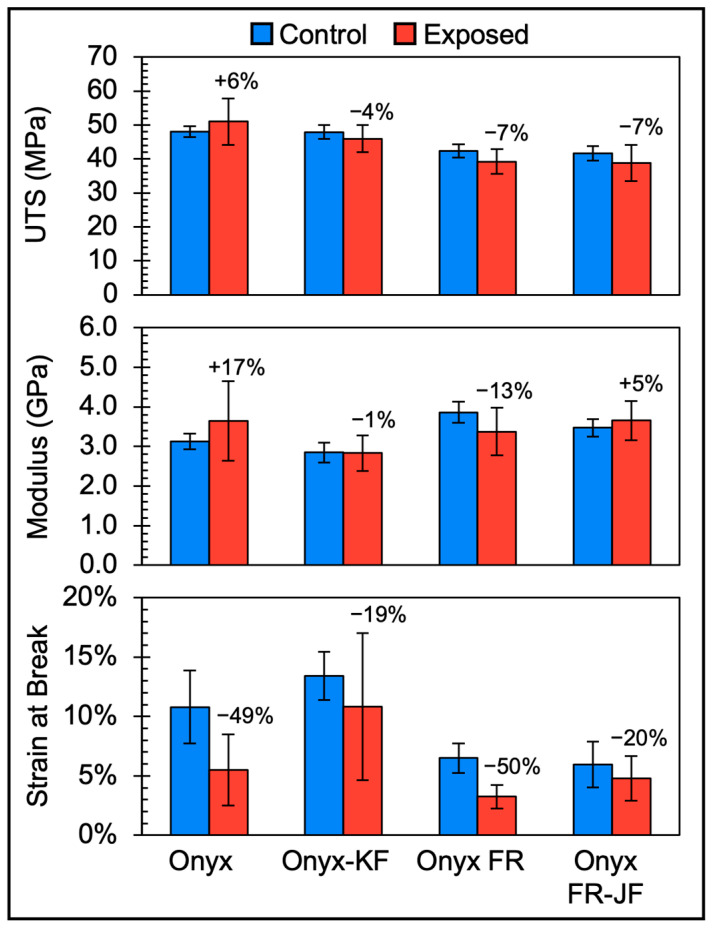
Tensile property bar charts comparing Control samples (blue, left column) and Exposed samples (red, right column) of short carbon fiber composite materials Onyx, Onyx-KF, Onyx FR, and Onyx FR-JF. Ultimate Tensile Strength—UTS, Modulus, and Strain at Break are shown for each sample type. Data points are the mean of 5 sample replicates. Error bars represent ±1 SD of the mean.

As seen in Figure 3, the conditioned samples have a lower modulus and lower ultimate tensile stress as compared to dry samples due to the known property dependence of moisture in Onyx and polyamides in general [14,15,16,17,18,19]. Exposed samples generally exhibit an increase in modulus, increase in UTS, and reduction in strain at break compared to the Control samples. Figure 4 highlights the tensile properties of all short fiber composite samples tested in this study.

As shown in Figure 4, Onyx exhibited increases in UTS and modulus (+6% and +17%, respectively), and a decrease in strain at break (−49%). Onyx-KF, the Onyx samples with a protective coating, exhibited decreases in UTS, modulus, and strain at break (−4%, −1%, and −19%, respectively). These results suggest that the Krylon Fusion All-in-one coating reduced the degree of embrittlement due to environmental exposure on Onyx samples.

Onyx FR exhibited decreases in UTS, modulus, and strain at break (−7%, −13%, and −50%, respectively). Onyx FR-JF, the Onyx FR samples with a protective coating, exhibited decreases in UTS and strain at break (−7 and −20%, respectively), and an increase in modulus (+5%). Similar to Onyx-KF, the JetFlex White Polyurethane Primer coating of Onyx FR-JF reduces the degree of embrittlement due to exposure on Onyx FR samples.

Overall, Exposed short fiber composite samples exhibited a 0–7% decrease in average UTS (93–100% retention of Control UTS). This is above the 70% property retention limit that UL requires for the tensile strength component of an f2 outdoor suitability rating. The Onyx and Onyx FR short fiber composite samples are expected to have some level of resistance to UV degradation due to the reduced penetration depth of UV light. The average moduli of the Exposed samples appear to remain largely unchanged when factoring in variation of the data, with the exception of Onyx, which appears to stiffen—consistent with embrittlement. The average strains at break of Exposed samples generally decreased as compared to the control. The uncoated samples (Onyx and Onyx FR) exhibited ~50% decreases in average strain at break while the coated samples (Onyx-KF and Onyx FR-JF) exhibit ~20% decreased in average strain at break. While the presence of coatings appears to reduce the effects of environmental exposure on the tensile strain at break, the variances are larger as compared to the uncoated Exposed samples. The reason for the increase in variance could be due to factors such as a possible inconsistency in coating thickness.

### 3.2. Tensile Properties of Continuous Fiber Composites

**Figure 5:**
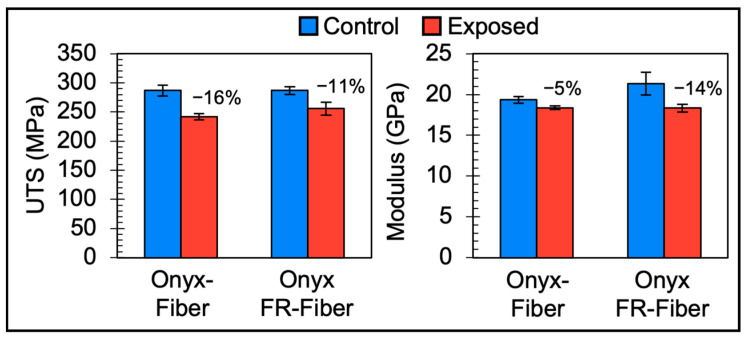
Tensile property bar charts comparing materials of construction (Onyx-Fiber, Onyx FR-Fiber), Control (blue, left columns), and Exposed (red, right columns) data for two different properties (Ultimate Tensile Strength—UTS and Modulus). Data points are the mean of 5 sample replicates. Error bars represent ±1 SD of the mean.

Composite parts reinforced with continuous carbon fiber offer an order of magnitude improvement in strength and stiffness over the short fiber composite parts, making them an excellent candidate for high-load, end-use applications. Therefore, the tensile properties of continuous fiber composite parts were investigated to better understand the environmental stability of Markforged composite parts. Figure 5 highlights the UTS and modulus of all continuous fiber composite samples tested in this study.

Following accelerated environmental exposure, Onyx-Fiber and Onyx FR-Fiber exhibited reductions in both UTS (16% and 11%, respectively) and tensile modulus (5% and 14%, respectively). Some degradation is expected due to an accumulation of moisture at the PA6-CCF interface, thereby weakening interfacial bonding [9]. However, even after environmental exposure, the samples maintained 5× the UTS of non-reinforced samples by adding only 9 vol% of continuous carbon fiber. Additional data on properties of Onyx FR-Fiber with environmental conditioning can be found in an upcoming report from the National Center for Advanced Materials Performance (NCAMP).

### 3.3. Flexural Properties of Short Fiber and Continuous Fiber Composites

Flexural properties are also of interest for end-use applications requiring knowledge of part anisotropy and the behavior of the materials under bending loads. This is of specific interest in the case of environmental exposure that predominantly affects the surface of the parts where the highest tensile and compressive forces are experienced during the test.

**Figure 6:**
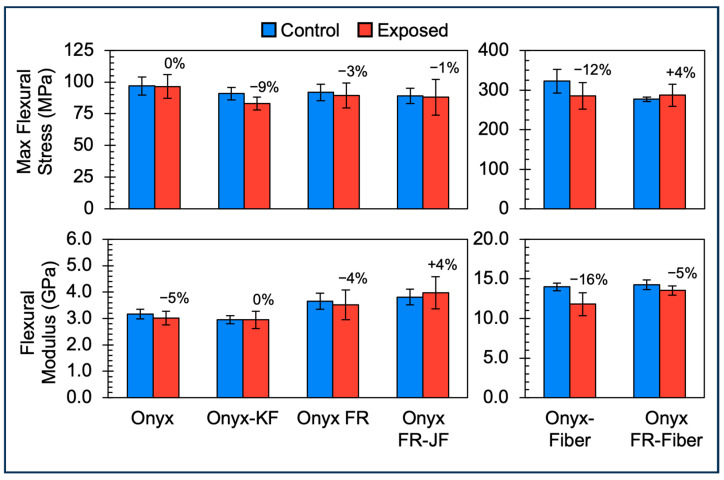
Flexural property bar charts comparing materials of construction (Onyx, Onyx-KF, Onyx FR, Onyx FR-JF, Onyx-Fiber, Onyx FR-Fiber), Control (blue, left columns), and Exposed (red, right columns) data for two different properties (Maximum Flexural Stress and Flexural Modulus). Data points are the mean of 5 sample replicates. Error bars represent ±1 SD of the mean. Note that the y-axis scale of the Onyx-Fiber and Onyx FR-Fiber samples is larger than the short fiber composite samples.

Figure 6 shows that Exposed short fiber composite Onyx samples exhibited no change in maximum flexural stress and a 5% decrease in flexural modulus as compared to Control samples. This is different from the tensile properties of Exposed Onyx samples, which saw general embrittlement with increases in UTS (+6%) and tensile modulus (+17%). This result is surprising, as any embrittlement would be expected to be detected by flexural testing as the technique is sensitive to material changes at the sample surface. This result is consistent with a decrease in the compressive strength of Onyx after exposure, as a flexural sample has a gradient of tensile and compressive stress and the tensile properties were shown to increase. Onyx-KF, Onyx FR, and Onyx FR-JF exhibited a 1–9% decrease in maximum flexural stress and a 0–4% decrease in flexural modulus. These values are in line with the changes seen for the respective tensile properties.

Similar to the short fiber composite samples, Onyx FR-Fiber samples exhibited 0% and 5% decreases in maximum flexural stress and flexural modulus, respectively, as compared to Control samples. Onyx-Fiber samples exhibited 12% and 16% decreases in maximum flexural stress and flexural modulus, respectively, as compared to Control samples. 

Overall, Exposed short fiber composite samples and continuous fiber composites samples maintained 84–100% of the maximum flexural stress of the Controls—above the 70% property retention limit that UL requires for the flexural strength component of an f2 outdoor suitability rating. Further, the maximum flexural stress values of continuous fiber composite samples were nearly 3× the maximum flexural stress of short fiber composite Onyx and Onyx FR samples. Higher loadings of fiber would expect to bring higher maximum flexural stress.

### 3.4. Impact Strength of Short Fiber and Continuous Fiber Composites

**Figure 7:**
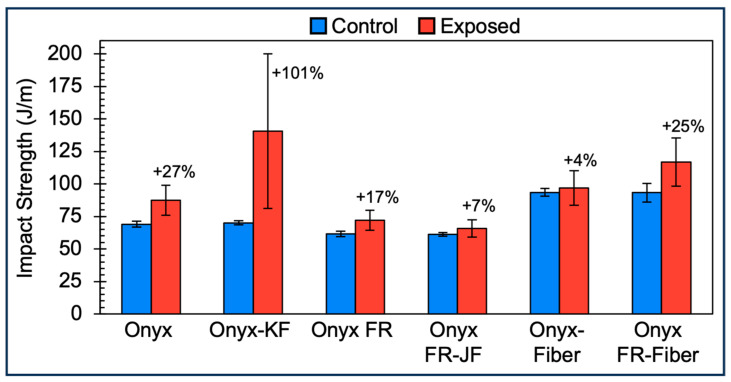
Impact strength bar chart comparing between materials of construction (Onyx, Onyx-KF, Onyx FR, Onyx FR-JF, Onyx FR Fiber, Onyx w/ Fiber), Control (blue, left columns), and Exposed (red, right columns) data. Data points are the mean of 5 sample replicates. Error bars represent ±1 SD of the mean.

Impact strength of short fiber and continuous fiber composite samples was tested, as shown in Figure 7.

The impact strength of Exposed samples increased 4–101% as compared to Controls. These increases in impact strength appear to contradict the reduction in toughness exhibited by the tensile results. In addition, the variance of the Exposed sample impact strengths appears to be greater than that of the Control samples. The increase in variance is consistent with the sensitivity of a notched impact technique to the presence of surface defects such as micro cracking, which were possibly present after exposure. Onyx-KF exhibited a high average increase in impact strength in addition to a high variation, which could be due to factors including a possible inconsistency in coating thickness at the notch and a potentially lower moisture vapor transmission rate with the presence of the coating. As for the increase in impact strength for all Exposed material samples, it is likely that this is a result of greater moisture retention during the environmental exposure processes.

After exposure, all samples were dried for 48 h at 75 °C prior to testing. Izod impact samples were thicker than tensile and flexural samples (8.1 mm versus 3.6 mm) and thus more likely to retain moisture. Moisture testing was performed post-testing on Onyx Izod impact samples upon receipt from the NRTL facility. First, we found that an Onyx Control Izod sample exhibited a higher moisture level (1.03% by weight) than Onyx Control tensile and flexural samples (<0.2% by weight). Next, we found that an Onyx Exposed Izod sample had a higher moisture level (1.56% by weight) than an Onyx Control Izod sample (1.03% by weight). The higher moisture level in exposed samples is consistent with the increased impact strength values. Therefore, Exposed Izod samples for all materials are expected to have a higher moisture content, supported by the increased impact strength values as shown in Figure 7. In addition, all Izod samples, given their higher thickness than tensile samples, are expected to retain more moisture than the tensile and flexural samples and are more likely to exhibit a toughening behavior with the absorbed moisture. Given that the testing aims to mimic outdoor environments, it is expected that end-use parts would have absorbed moisture and thus have higher impact values.

### 3.5. Visual Differences between Unexposed and Exposed Samples

**Figure 8:**
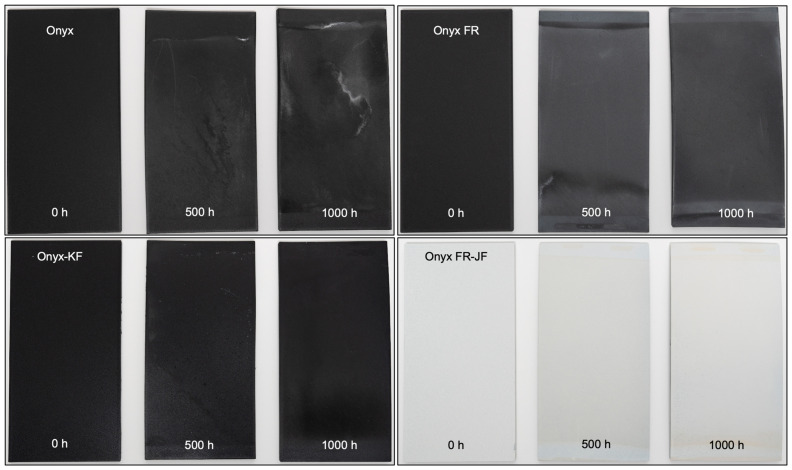
Visual appearance of test plaques after 500 h and 1000 h of environmental exposure for Onyx, Onyx-KF, Onyx FR, and Onyx FR-JF coating.

Surface finish and appearance are important for customers considering materials in end-use applications and are often important elements for end-use parts. A matte black, smooth surface finish is a hallmark of a Markforged part and any visual differences over time may be important to customers. The visual differences of Onyx, Onyx-KF, Onyx FR, and Onyx FR-JF before, during (500 h), and after (1000 h) environmental exposure are shown in Figure 8. A white residue is apparent on the surface of Onyx and Onyx FR plaques. This residue is caprolactam, a small molecule within the PA6 blend that acts as a plasticizer in the Onyx and Onyx FR base plastics. The caprolactam residue and visual defects are less apparent on Onyx-KF and Onyx FR-JF plaques. Thus, the coatings help maintain a visual appearance similar to the Control samples even after environmental exposure for 1000 h.

### 3.6. Role of Residual Caprolactam in Stabilization of Onyx

The filled PA6 blend polymers used in this study contain ~5–6 wt% caprolactam. PA6 can be created from the ring-opening polymerization of an ε-caprolactam monomer, most commonly via a hydrolytic polymerization [28,29]. The reaction runs to an equilibrium of ~90% yield, with a remaining ~10 wt% composed of ε-caprolactam monomer (8 wt%), and oligomeric PA6 (2 wt%) [29,30]. The remaining caprolactam fraction is advantageous for melt processing, printing (as a viscosity modifier), and also for toughening (as a plasticizer) an otherwise brittle short carbon-fiber-filled PA6. The caprolactam content of each set of Control and Exposed Onyx tensile samples in this study were found to be 5.3 ± 0.3 wt% and 3.7 ± 1.6 wt%, respectively. The reduction in caprolactam content from 5.3 wt% to 3.7 wt% results in an increase in UTS from ~48 MPa to ~51 MPa. This finding highlights how the residual caprolactam not only aids in processing and part creation, but also mechanical property stabilization under environmental exposure.

## 4. Conclusions

This study involved the comparison of mechanical properties, specifically tensile, flexural, and impact, and visual properties before and after accelerated environmental exposure using ASTM G155 Cycle 1 conditions and a Xenon-arc energy source. We find:

- Tensile strength, maximum flexural stress, and impact strength of Exposed samples all retained greater than 80% of the properties of their Control counterparts—above the 70% minimum tensile, flexural, and impact strength retention requirements posed by UL 746C f1/f2 outdoor suitability testing. Thus, end-use parts that require stable mechanical properties after environmental exposure can be created from short fiber and continuous fiber composite Onyx and Onyx FR.
- Continuous fiber composite samples tested in this study retained 3×–5× mechanical strengths over short fiber composite samples; continuous fiber composite samples with higher loadings would maintain higher strengths.
- Coatings are beneficial in maintaining visual appearance, as shown through the use of Krylon Fusion All-in-one spray paint on Onyx and JetFlex White Polyurethane Primer on Onyx FR.
- Residual caprolactam monomer aids in mechanical property stabilization of Onyx subject to environmental exposure and is likely one reason why Onyx maintains a high level of resistance to the effects of environmental exposure.
- The moisture absorbed during exposure likely played a role in impact strength improvement due to property dependence of absorbed moisture in polyamides.

## Figures and Tables

**Table 1:** Test coupon types and dimensions.

Sample Type	Sample Dimensions		
	Length (mm)	Width (mm)	Thickness (mm)
Tensile	ASTM Type I Dogbone		3.6
Izod Impact	64	13	8.1
Flexural	64	13	3.6
Visual	150	75	3.6
Tensile Fiber	178	25	3.6

**Table 2:** Fiber print parameters for continuous fiber composite samples.

Fiber Sample Type	Fiber Layer Group Orientation (°)	Fiber Layer Numbers	
		Lower Layer Group	Upper Layer Group
Tensile	0°	5–8	22–25
Flexural	0°	5–8	22–25
Izod Impact	−45°, 0°, 45°, 90°	5–8	58–61

## Data Availability

The data presented in this study are available on request from the corresponding author. The data are not publicly available due to involving proprietary materials.
